# Study of tRNA-Derived Fragment tRF-20-S998LO9D in Pan-Cancer

**DOI:** 10.1155/2022/8799319

**Published:** 2022-05-05

**Authors:** Jinqi Ma, Fengxia Liu

**Affiliations:** Department of Blood Transfusion, The Third Xiangya Hospital of Central South University, Changsha 410013, China

## Abstract

**Objective:**

The purpose is to study the effect of tRNA-derived fragments (tRFs) on pan-cancer through bioinformatics.

**Methods:**

The expression information of tRF-20-S998LO9D, a type of tRF-5, was retrieved through MINTbase in pan-cancer and verified by qPCR. We preliminarily explored the effect of tRF-20-S998LO9D on cell proliferation in breast cancer and lung cancer cell lines. Then an online KM-plotter provided by OncotRF was used to discover the prognostic significance. GO/KEGG analyses were executed to predict the potential mechanism of tRF-20-S998LO9D in cancer.

**Results:**

We found that tRF-20-S998LO9D was highly expressed in a variety of cancers like breast invasive carcinoma, head and neck squamous cell carcinoma, kidney renal clear cell carcinoma, lung squamous cell carcinoma, pheochromocytoma and paraganglioma, and uterine corpus endometrial carcinoma. Inhibition of tRF-20-S998LO9D led to reduced cell proliferation in breast cancer (MCF-7) and lung squamous cell carcinoma (SK-MES-1) cells. Elevated tRF-20-S998LO9D indicated poor prognosis in a variety of cancers. tRF-20-S998LO9D might be involved in multiple cancer-related pathways.

**Conclusion:**

We concluded that tRF-20-S998LO9D was upregulated and negatively correlated with prognosis of a variety of cancers. It may be a potential cancer-promoting marker in pan-cancer.

## 1. Introduction

The genesis and evolution mechanisms of cancer have always been a key focus of research. Considerable research highlights the regulatory mechanisms of noncoding RNAs in the development of diseases [1]. These RNAs usually do not code proteins and are divided into long noncoding RNAs (lncRNA) and small noncoding RNAs (sncRNA) based on their length. Studies have revealed the significance of lncRNA and multiple sncRNAs, like microRNA, small interfering RNA, and transfer RNA (tRNA), in the occurrence and development of cancer. For example, Kong et al. found that lncRNA-CDC6 could function as competitive endogenous RNA (ceRNA) via directly sponging of microRNA-215, which further regulate the expression of CDC6 in breast cancers [2]. Let-7, a microRNA, was downregulated in breast, colon, and lung cancers and proven to prevent tumor development by repressing RAS or MYC [3, 4]. With the advance in next-generation sequencing and bioinformatics technology, a new class of small noncoding RNAs derived from tRNAs has raised great concerns.

These tRNA-derived small RNAs are called tRFs (tRNA-derived fragments). Since they were initially discovered in the 1970s, tsRNAs were once considered nonfunctional in biological processes [5]. However, there is increasing evidence that tsRNAs can participate in different molecular and physiological processes in recent years. tRFs are highly conservative, and their biogenesis require a precise site-specific cutting [6]. Based on the cleavage position of tRFs, they are generally categorized as tRF-1, tRF-5, tRF-3, tRF-2, and tiRNA [7]. Dysregulation of tRFs has been reported to participate in tumor-promoting or suppressive activities in several cancers [8, 9].

tRF-5s with 14-30 nt in length begin at the 5′ ends of the mature tRNAs and extend into the D-loop or the stem region between the D-loop and anticodon loop of the tRNA [10]. tRF-5s have been considered to play important roles in various pathophysiological processes. tRF-20-S998LO9D, a tRF-5 derived from chromosome 1 tRNA86^ArgTCT^, was identified as a potential prognostic factor in patients with head and neck squamous cell carcinoma in previous reports [11]. But its significance in pan-cancer remains unknown. Based on the advancement of public data platforms and bioinformatics technology, pan-cancer analysis methods provide the potential to identify the common characteristics among cancers.

Here, for the first time, we preliminarily explored the expression and prognosis significance of tRF-20-S998LO9D in pan-cancer using MINTbase and OncotRF database. Cell proliferation assay and the potential molecular mechanism of S998LO9D predicted by TargetScan and DAVID Bioinformatics Resources further suggested the cancer-promoting effect.

## 2. Methods

### 2.1. Retrieval of tRF Expression Data from MINTbase

The MINTbase v2.0 database (https://cm.jefferson.edu/MINTbase/) was employed to retrieve the tRF expression status [12]. MINTbase unique ID (“tRF-license plate”) was used for tRF nomenclature. The expression information was outputted in tabular form including the tRF's normalized abundance (in RPM) and the tissue type. The expression differences of tRF-20-S998LO9D in pan-cancer and matched control tissues were organized and visualized by the R project.

### 2.2. Clinical Tissues

Cancer and para-cancer tissues of 15 BRCA (breast invasive carcinoma), HNSC (head and neck squamous cell carcinoma), KIRC (kidney renal clear cell carcinoma), LUSC (lung squamous cell carcinoma), PCPG (pheochromocytoma and paraganglioma), and UCEC (uterine corpus endometrial carcinoma) were obtained at the Third Xiangya Hospital. Fresh tissues were preserved in liquid nitrogen. All patients provided informed consent.

### 2.3. Cell Culture and Viability Assay

Human breast tumor cell line MCF-7 and lung squamous cell carcinoma cell line SK-MES-1 were gifts from Cancer Institute of Central South University. Cancer cells were cultured according to the recommended protocols. MCF-7 and SK-MES-1 were transfected with small interfering RNA (GTCCATTGCGCCACAGAGA) using Lipofectamine™ 3000 (Invitrogen, MA, USA). For viability assay, cells were incubated in 96-well plates (2 × 10^4^ cells/well) for 48 hours. Cell Count Kit-8 reagent (Sigma Aldrich, St Louis, Missouri; 10 *μ*L) was added to each wells, followed by incubation for 4 hours. Absorbance of the solution was measured at 450 nm using an ELx800 microplate reader (Winooski, Vermont, USA).

### 2.4. RNA Extraction and qPCR

We extracted total RNA from clinical samples with TRIzol (Invitrogen, USA) based on the instruction manual. RNA samples were quantified by NanoDrop ND-1000 (NanoDrop, USA). Reverse transcription was carried out with PrimeScript RT Reagent Kit (Takara, China). qRT-PCR was executed with ViiA 7 Real-Time PCR System (Applied Biosystems). The 2^-*ΔΔ*Ct^ method was used for calculating relative expression levels of tRF-20-S998LO9D. The forward primer sequence for tRF-20-S998LO9D is 5′-TCTCTGTGGCGCAATG-3′, and the reverse primer sequence is GGTCCAGTTTTTTTTTTTTTTTGTC.

### 2.5. tRF Prognostic Data Retrieved in the OncotRF Database

An online KM-plotter provided by OncotRF (http://bioinformatics.zju.edu.cn/OncotRF/), which collated RNA sequencing data and prognostic information of patients from TCGA, was used to discover the prognosis significance of tRF-20-S998LO9D in pan-cancer [13]. Patients were classified into low-expression or high-expression groups according to the median tRF level. The OS and DFS information of tRF-20-S998LO9D in different cancers were summarized.

### 2.6. Functional Analysis of tRF-20-S998LO9D by Bioinformatics

TargetScan algorithm, which is based on searching for the 8mer and 7mer sites that match the seed region of noncoding RNA, was used to predict biological targets of the tRF-20-S998LO9D [14]. DAVID Bioinformatics Resources 6.8 (https://david.ncifcrf.gov/home.jsp), a functional annotation website, was used for GO and KEGG function enrichment analysis [15]. The prediction results were visualized by GraphPad Prism 8.0.1 and Cytoscape 3.7.2.

### 2.7. Statistical Analysis

Shapiro-Wilk tests were used to assess data normality. For normally distributed data, a two-tail unpaired Student's *t*-test was used. For nonnormally distributed data, a nonparametric test (Mann-Whitney test) was taken. *P* value < 0.05 was considered significant.

## 3. Results

### 3.1. Aberrant Expression of tRF-20-S998LO9D in Pan-Cancer

**Figure 1:**
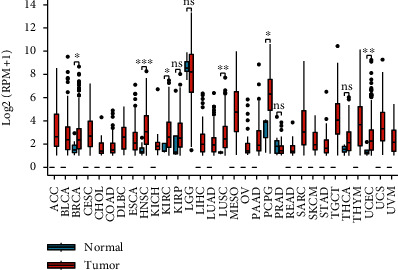
Expression of tRF-20-S998LO9D in pan-cancer. BLCA: bladder cancer; CESC: cervical cancer; CHOL: bile duct cancer; COAD: colon cancer; DLBC: large B-cell lymphoma; ECSA: esophageal cancer; KIRP: kidney papillary cell carcinoma; LGG: lower grade glioma; LIHC: liver cancer; OV: ovarian cancer; PAAD: pancreatic cancer; PRAD: prostate cancer; READ: rectal cancer; SARC: sarcoma; SKCM: melanoma; STAD: stomach cancer; TGCT: testicular cancer; UVM: ocular melanomas (ns: *P* > 0.05, ∗*P* < 0.05, ∗∗*P* < 0.01, ∗∗∗*P* < 0.001, ∗∗∗∗*P* < 0.0001).

**Figure 2:**
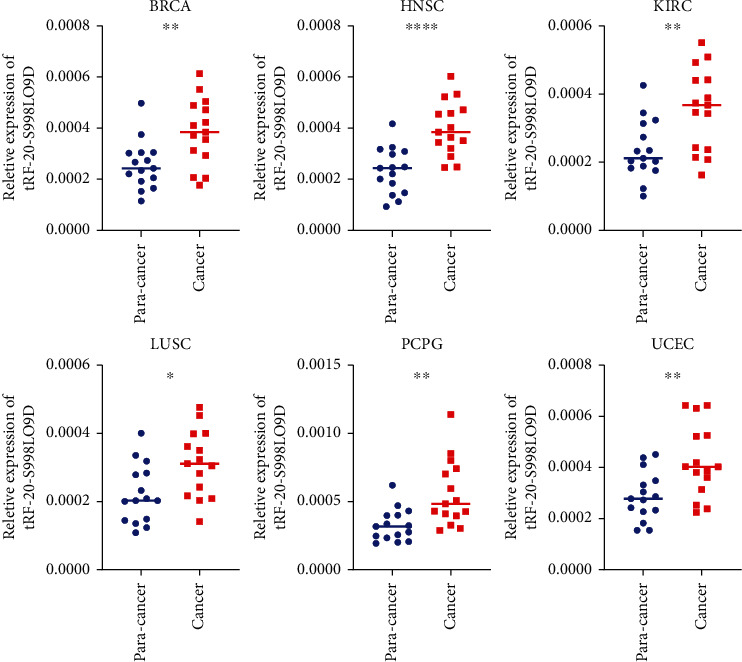
Relative expression of tRF-20-S998LO9D in BRCA, HNSC, KIRC, LUSC, PCPG and UCEC by qPCR.

The information on the expression level of tRF-20-S998LO9D was retrieved in 31 types of cancers (Figure 1). Among them, ten cancer datasets contained normal controls (sample size ≥ 3). tRF-20-S998LO9D was significantly highly expressed in a variety of cancers like BRCA (*P* = 0.0397), HNSC (*P* = 0.0008), KIRC (*P* = 0.0153), LUSC (*P* = 0.0026), PCPG (*P* = 0.0105), and UCEC (*P* = 0.0045). Unfortunately, normal controls were missing in many cancer datasets. But overall tRF-20-S998LO9D expression showed a trend of an increased level in pan-cancer tissues. To further confirm the dysregulation status of tRF-20-S998LO9D in pan-cancer, we collected clinical specimens from patients with BRCA, HNSC, KIRC, LUSC, PCPG, and UCEC. We found a significant upregulation of expression of tRF-20-S998LO9D in all these cancers (*P* value was 0.0048, <0.0001, 0.0044, 0.0123, 0.0024, and 0.0047, respectively) (Figure 2).

### 3.2. tRF-20-S998LO9D Promoted the Proliferation of Tumor Cells In Vitro

**Figure 3:**
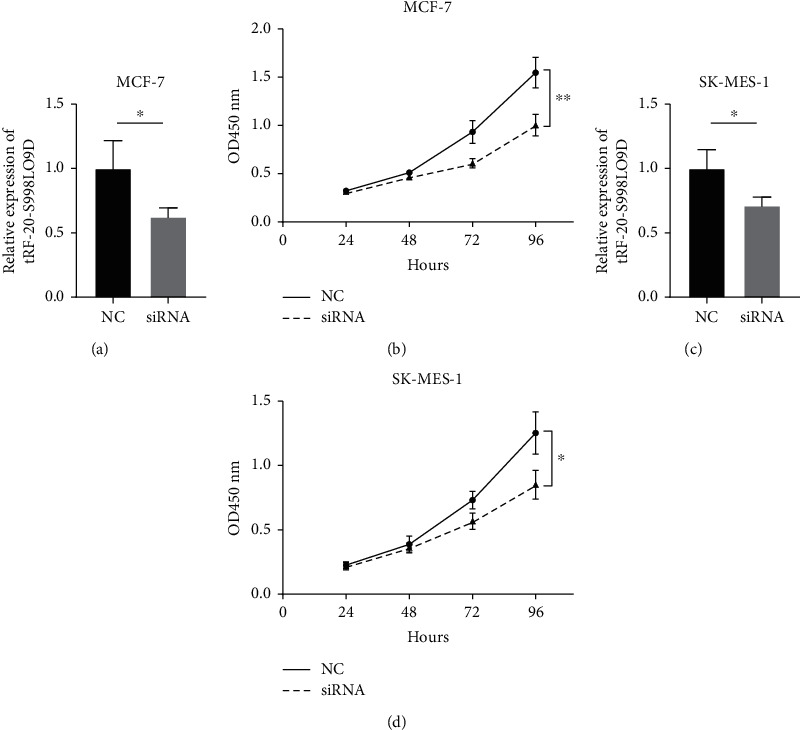
Role of tRF-20-S998LO9D in tumor cell proliferation regulation. (a) After siRNA transfection, tRF-20-S998LO9D expression in MCF-7 cells was inhibited (*P* = 0.0449); (b) inhibition of tRF-20-S998LO9D reduced the proliferation of MCF-7 breast cancer cells (*P* = = 0.0086); (c) siRNA transfection successfully inhibited the expression of tRF-20-S998LO9D in SK-MES-1 lung squamous cell carcinoma cells (*P* = 0.0368); (d) inhibition of tRF-20-S998LO9D reduced the proliferation of SK-MES-1 cells (*P* = 0.0247).

Preliminary exploration of the function of tRF-20-S998LO9D was conducted in breast and lung cancer, two of the most common cancers in adults. Firstly, we constructed cell lines with lower expression of tRF-20-S998LO9D in breast cancer (MCF-7) and lung squamous cell carcinoma (SK-MES-1) cells. The successful transfection was observed by qPCR in both cell lines (*P* value was 0.0449 and 0.0368, respectively) (Figures 3(a) and 3(c)). As shown in cell proliferation curve, inhibition of tRF-20-S998LO9D in MCF-7 cells led to decreased cell proliferation rate, and the OD value of the NC group was about 1.5 times that of the siRNA group at 96 h (*P* = 0.0086) (Figure 3(b)). A consistent trend was observed in SK-Mes-1 cells (*P* = 0.0086) (Figure 3(d)).

### 3.3. The Prognostic Significance of tRF-20-S998LO9D in Pan-Cancer

**Figure 4:**
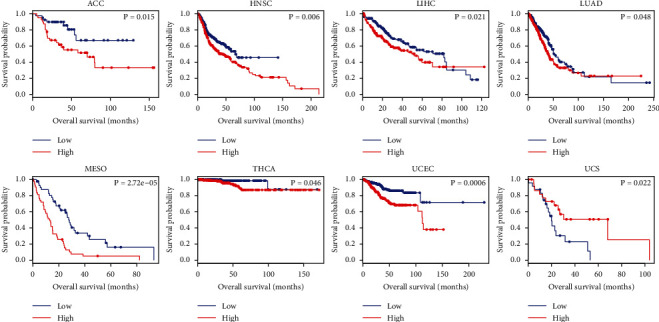
The relationship between tRF-20-S998LO9D and OS in ACC, HNSC, LIHC LUAD, MESO, THCA, UCEC, and UCS.

**Figure 5:**
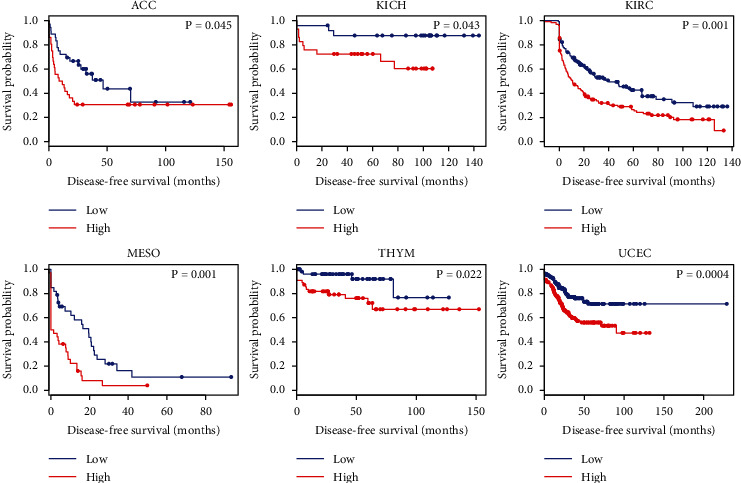
The relationship between tRF-20-S998LO9D and DFS in ACC, KICH, KIRC, MESO, THYM, and UCEC.

To evaluate the prognostic value, we assessed the correlation between abnormally expressed tRF-20-S998LO9D and clinical outcome with KM-plotter. Highly expressed tRF-20-S998LO9D was significantly associated with poor overall survival (OS) in ACC (adrenocortical carcinoma), HNSC, LIHC (liver hepatocellular carcinoma), LUAD (lung adenocarcinoma), MESO (mesothelioma), THCA (thyroid carcinoma), UCEC, and UCS (uterine carcinosarcoma) (Figure 4). In ACC, KICH (kidney chromophobe), KIRC, MESO, THYM (thymoma), and UCEC, tRF-20-S998LO9D was significantly associated with poor disease-free survival (DFS) (Figure 5).

### 3.4. Function Prediction of tRF-20-S998LO9D

**Figure 6:**
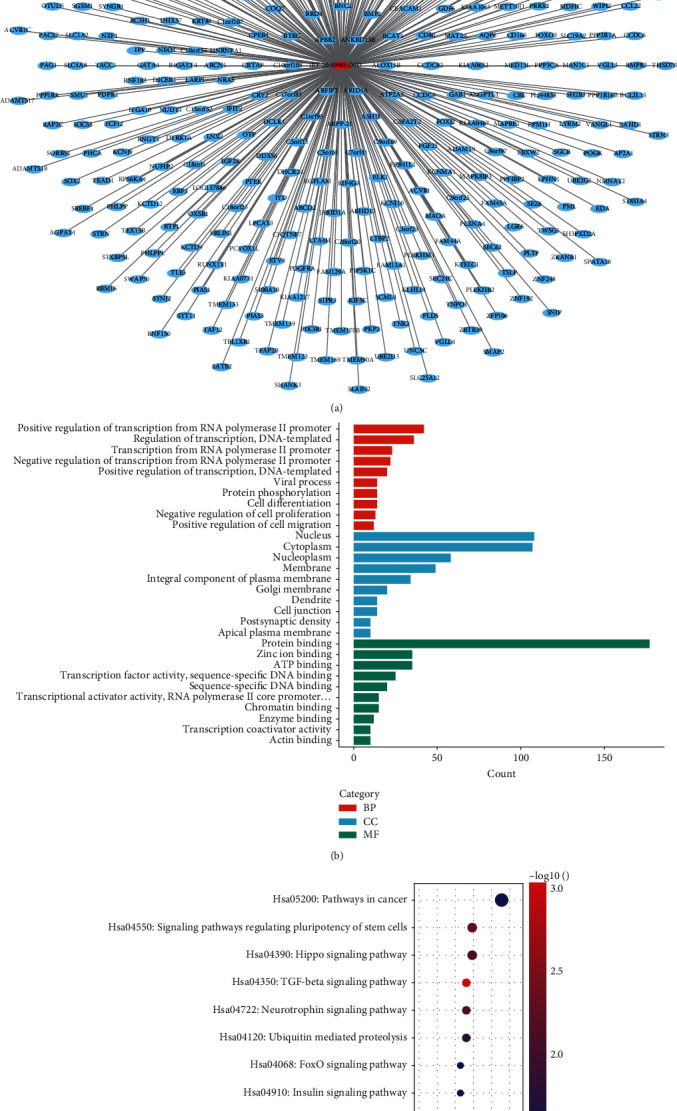
Functional analysis of tRF-20-S998LO9D. (a) Target genes of tRF-20-S998LO9D. (b) GO enrichment analysis of tRF-20-S998LO9D. (c) KEGG pathway analysis of tRF-20-S998LO9D.

TargetScan algorithms were used to predict the potential function of tRF-20-S998LO9D. 369 conserved targets were obtained. The target genes were shown in Figure 6(a). DAVID databases were used for functional enrichment analysis of putative target genes of tRF-20-S998LO9D. Gene ontology (GO) enrichment analysis indicated that 89 GO terms were enriched (*P* < 0.05) for target genes of tRF-20-S998LO9D. The most enriched terms of target genes of tRF-20-S998LO9D were “positive regulation of transcription from RNA polymerase II promoter” and “regulation of transcription, DNA-templated” in biological process (BP) category, “nucleus” and “cytoplasm” in cellular component (CC) category, and “protein binding” and “zinc ion binding” in molecular function (MF) category (Figure 6(b)). 13 pathways were obtained in the Kyoto Encyclopedia of Genes and Genomes (KEGG) pathway analysis, and the ten most significant were displayed in Figure 6(c). The most enriched pathways like pathways in cancer, signaling pathways regulating pluripotency of stem cells, and Hippo signaling pathway have been reported to participate in the genesis and evolution of cancer. Other enriched pathways include TGF-Beta signaling pathway, ubiquitin-mediated proteolysis, FoxO signaling pathway, non-small-cell lung cancer, melanoma, endometrial cancer, central carbon metabolism in cancer, and chronic myeloid leukemia which are also considered to be closely related to the occurrence and development of tumors.

## 4. Discussion

Although dysregulation of tRFs has been described in cancers, their commonalities remain poorly investigated. Here, we investigated the expression of tRF-20-S998LO9D in pan-cancer, as well as its prognostic potential and downstream pathways.

Our results showed that tRF-20-S998LO9D was upregulated in a variety of cancers. Since the MINTbase v2.0 only comprises tRFs with an abundance ≥ 1.0 RPM, tRFs with expression levels that are either zero or just under the threshold are not recorded. On the other hand, the overall read-counts of tRFs are relatively low, and the population of normal controls is relatively small. These may lead to limited available normal control samples. Despite these limitations, it is reasonable to conclude that dysregulation of tRFs may be a universal phenomenon in pan-cancer.

Unlimited proliferation is an important sign of malignancy. Tumor microenvironment, dysregulation of oncogene/oncogene, epigenetic abnormalities, and many other factors have been considered to be associated with cancer cell proliferation [16–19]. The study of proliferation characteristics can improve our understanding of the cell cycle and the pathogenesis of malignant tumors. In the previous studies, tsRNAs have been found to regulate tumor cell proliferation and colony formation in various tumors such as lung cancer, colorectal cancer, prostate cancer, breast cancer, ovarian cancer, leukemia, and lymphoma [7, 9, 20, 21]. However, the role of tRF-20-S998LO9D in tumor proliferation remains unknown. Our study suggested a positive correlation between tRF-20-S998LO9D expression level and tumor cell proliferation. These phenomenon indicates that tRF-20-S998LO9D may be involved in the regulation of the progression of multiple tumor types.

Numerous studies have indicated that tRFs may be ideal biomarkers for the prognosis of cancers of the breast, ovaries, lung, prostate, colorectum, and other organs [22–26]. The prognostic value of tRF-20-S998LO9D in HNSC patients has also been confirmed in previous reports [11]. Our study further illustrated the prognostic significance of tRF-20-S998LO9D in pan-cancer. This phenomenon suggests that tRF-20-S998LO9D may be extensively involved in the progression or treatment response of a variety of tumors. To date, tsRNAs dysregulation in serum, urine, sperm, and other body fluids have been identified in cancer and some other diseases [21, 27, 28]. These indicate that tsRNAs can be taken as a tumor circulation molecule and thus suggest the prospect of tsRNAs as clinical noninvasive biomarkers.

Although the biological roles of tRFs have been revealed in various pathophysiological processes, their potential mechanisms are largely unknown and require further elucidation. In general, the role of tsRNAs can be summarized as RNA silencing, translation regulation, and epigenetic regulation [29]. Studies have indicated that tRFs can interact with RNA-binding proteins and regulate gene silencing by directly targeting mRNAs like miRNAs [30]. Gu et al. reported that tRF-20-S998LO9D might bind to eIF4B and SRSF1 according to predictions from RNA-Binding Protein DataBase (RBPDB) [11]. On the other hand, the roles of eIF4B and SRSF1 in cancer have been widely elucidated. These suggest the potential of tRF-20-S998LO9D in tumorigenesis and development.

KEGG pathway prediction showed that tRF-20-S998LO9D might be involved in cancer hallmark pathways. For instance, Hippo signaling has been demonstrated to play a crucial role in cell proliferation and contribute to cancer progression [31]. Dysregulation of the TGF-*β* signaling pathway can promote tumorigenesis, including metastasis and chemoresistance [32]. The proteolysis mediated by ubiquitin is vital for cell-cycle regulation through recognition, interaction, and ubiquitination or deubiquitination of key proteins. The abnormally high accumulation or illegitimate degradation of tumor suppressor proteins and oncoproteins results in tumorigenesis [33]. FoxO signaling pathways are also critical to cancer cell biology through implicating in cell differentiation, apoptosis, proliferation, DNA damage, and repair [34]. Altogether, these suggest the potential mechanism of tRF-20-S998LO9D in tumorigenesis and development.

We note a few limitations to this study. Firstly, due to the small sample size of available normal controls, differential expression data cannot be fully obtained in all cancer groups. Secondly, the underlying mechanism of tRF-20-S998LO9D in cancer has not been experimentally validated.

## 5. Conclusion

tRF-20-S998LO9D was dysregulated and associated with poor prognosis in a variety of cancers. It may act as a cancer-promoting biomarker in pan-cancer.

## Data Availability

The data used to support the findings of this study are available from the corresponding author upon request.
